# Polyphasic Characterization of Four *Aspergillus* Species as Potential Biocontrol Agents for White Mold Disease of Bean

**DOI:** 10.3390/jof8060626

**Published:** 2022-06-12

**Authors:** Osama O. Atallah, Yasser S. A. Mazrou, Mahmoud M. Atia, Yasser Nehela, Abdelrazek S. Abdelrhim, Maha M. Nader

**Affiliations:** 1 Department of Plant Pathology, Faculty of Agriculture, Zagazig University, Zagazig 44519, Egypt; usamaatia2@yahoo.com; 2 Business Administration Department, Community College, King Khalid University, Guraiger, Abha 62529, Saudi Arabia; ymazrou@kku.edu.sa; 3 Department of Agriculture Economic, Faculty of Agriculture, Tanta University, Tanta 31527, Egypt; 4 Department of Agricultural Botany, Faculty of Agriculture, Tanta University, Tanta 31511, Egypt; yasser.nehela@ufl.edu; 5 Department of Plant Pathology, Faculty of Agriculture, Minia University, El-Minya 61512, Egypt; abdelrazek.sharawy@mu.edu.eg; 6 Agricultural Microbiology Department, Faculty of Agriculture, Zagazig University, Zagazig 44519, Egypt; mahanaderdiab@gmail.com

**Keywords:** polyphasic characterization, *Aspergillus* spp., biological control, *Sclerotinia sclerotiorum*

## Abstract

The genus *Aspergillus* comprises several species that play pivotal roles in agriculture. Herein, we morphologically and physiologically characterized four genetically distinct *Aspergillus* spp., namely *A. japonicus*, *A. niger*, *A. flavus,* and *A. pseudoelegans*, and examined their ability to suppress the white mold disease of bean caused by *Sclerotinia sclerotiorum* in vitro and under greenhouse conditions. Seriation type of *Aspergillus* spp. correlates with conidiospores discharge as detected on the Petri glass lid. Members of *Nigri* section cover their conidial heads with hard shells after prolonged incubation. In addition, sporulation of the tested *Aspergillus* isolates is temperature sensitive as it becomes inhibited at low temperatures and the colonies become white. Examined *Aspergillus* spp. were neither infectious to legumes nor aflatoxigenic as confirmed by HPLC except for *A. flavus* and *A. pseudoelegans* which, secreted 5 and 1 ppm of aflatoxin B1, respectively. Co-inoculations of *Sclerotinia*’s mycelium or sclerotia with a spore suspension of *Aspergillus* spp. inhibited their germination on PDA at 18 °C and 28 °C, and halted disease onset on detached common bean and soybean leaves. Similarly, plants treated with *A. japonicus* and *A. niger* showed the highest survival rates compared to untreated plants. In conclusion, black *Aspergillus* spp. are efficient biocides and safe alternatives for the management of plant diseases, particularly in organic farms.

## 1. Introduction

The white mold pathogen, *Sclerotinia sclerotiorum* (Lib.) de Bary, infects a wide range of plants causing serious economic losses in yield and crop quality [1,2,3,4,5]. More than 600 host plants are subjected to attack by such necrotrophic pathogens [2,4,6]. Members of the Leguminosae family including common bean and soybean are among the heavily damaged hosts by *S. sclerotiorum* [1,2,5,7,8,9]. Initial infections occur via ascospores liberated from fully developed apothecia [3,10]. Symptomatic plants exhibit water-soaked lesions throughout the canopy covered by white fluffy mycelium under humid conditions. Black amorphous sclerotia are then formed among the woven mycelial mats on and in the host canopy [11]. The melanized rind of sclerotia provides protection against adverse environmental conditions including heat, drought, and hydrolytic enzymes [12,13,14]. Melanized sclerotia aid for over-wintering and initial propagation by generating new progeny of ascospores after carpogenic germination.

The pathogenic capability of *S. sclerotiorum* relies on several physical and biochemical factors including compound appressoria, cell-wall degrading enzymes (CWDEs), oxalic acid, and isothiocyanate hydrolase [4,6,15]. Such complexity made it difficult to breed for resistance against this necrotrophic plant pathogen [4]. In addition, the resistibility of *Sclerotinia’s* resting structures increases the pathogen longevity and renders management strategies unreliable. Fortunately, biological control strategies using *Coniothyrium minitans* and *Sporidesmium sclerotivorum* bioagents have provided efficient solutions for such aggressive pathogens due to their mycoparasitic capability for the pathogen’s sclerotia [16,17].

Biological control of plant pathogens has proven efficient against white mold disease of oilseed rape, cabbage, soybean, common bean, and other vegetables and field crops [18,19,20,21,22]. *Aspergillus* spp. showed efficient suppression against a broad range of plant pathogenic fungi, bacteria, and nematodes [23,24,25,26,27,28,29,30]. The suppressive effect of *Aspergillus* was explained by several mechanisms including direct mycoparasitism [31,32], indirect inhibitory secretome such as hydrolytic chitinase and β-glucanase enzymes [22,33], and organic acids [34,35,36,37].

The genus *Aspergillus* comprises versatile species of different morphological, biological, and ecological characters. Species of *Aspergillus* are widespread throughout the agricultural environment, growing in soil, decaying organic matter, plants, and animals. The diversity of *Aspergillus* species is correlated with their ability to utilize a wide variety of organic substrates and adapt well to a broad range of environmental conditions [38]. *A. niger*, for example, is a common soil fungus that can live in different types of soils across a pH range from a highly acidic to a strongly alkaline environment [39]. Similarly, *A. japonicus* is found in soil and has also been known to adapt to agricultural environments and farmed animals [40]. The positive contribution of *Aspergillus* species is not limited to their antagonistic potential. Numerous members of *Aspergillus* are plant endophytes as they concomitantly live within their host plants in a symbiotic relationship. The symbiont *Aspergillus* partner provides growth promotion for the host plants and protection against a wide array of plant pathogens [41,42,43,44]. Other members of *Aspergillus* spp. serve as bio-remediators for the agricultural environment by reverting hazardous chemical compounds into environmentally benign forms and minimizing the accumulation of heavy metals [45,46,47,48]. Several *Aspergillus* spp. have economic value for mass production of lipids, proteins, enzymes refined organic acids [49,50,51]. Therefore, *Aspergillus* is an important genus that comprises significantly valuable species with direct and indirect benefits to agriculture and industry.

This work was established to evaluate the biocontrol potential for diverse species of *Aspergillus* on white mold disease of legumes caused by *S. sclerotiorum* under lab and greenhouse conditions. The examined species were genetically identified, and their organic acid profile was characterized in our previous work [37]. As a continuation, the morphological, microscopic, mycotoxigenic, and pathological attributes were characterized as well to demonstrate variability among tested species. In addition, the biocontrol potential of *Aspergillus* spp. was demonstrated in vitro, and on white mold-infected bean under greenhouse conditions.

## 2. Materials and Methods

### 2.1. Fungal Isolates and Plant Materials

Four *Aspergillus* spp. were previously isolated and genetically identified based on internal transcribed spacer (ITS) and beta-tubulin partial sequences [37]. *Sclerotinia* *sclerotiorum* was previously isolated from common bean plants exhibiting typical symptoms of white rot disease and maintained on PDA (Difco, Sparks, MD, USA) medium. Air-dried sclerotia were stored at −20 °C for long-term storage.

Common bean (*Phaseolus* *vulgaris* cv. Giza 6), soybean (*Glycine max* [L.] Merr. cv. Giza22), cowpea (*Vigna unguiculata* cv. Dokki7), and tobacco (*Nicotiana benthamiana*) plants were grown in the greenhouse under natural sunlight at a temperature range of 20–26 °C. Leaves were excised from two-month-old plants and used for the inoculation test.

### 2.2. Morphological Characterization of Aspergillus Isolates

Characterization procedures were carried out based on methods recommended by [52,53,54,55]. Spores suspension of *Aspergillus* spp. was prepared and adjusted to 10^6^ cfu/mL^−1^ as previously shown [37,56]. Spore suspension of each fungal isolate was used for three-point inoculation on MEA (Oxoid, Basingstoke, Hampshire, England) and PDA. Fungal isolates were incubated at 28 °C for seven days in the dark. Morphological characters of fungal colonies were the basis of discrimination among the isolates. Sclerotial production was examined by the naked eye and using an Olympus SZ61 Stereo Microscope 0.67–4.5×, Tokyo, Japan. The possible production of cleistothecia was tested on PDA and MEA after six weeks of inoculation using microscopy as well. Fungal specimens were mounted in lactic acid 60% and stained with lactophenol cotton blue for microscopy. Ethanol was used for mounting to prevent air bubbles and remove excessive conidia. Microscopic observations and digital imaging for fungal spores and spore-bearing structures were performed using a Leica DM500 compound microscope outfitted with an ICC50 HD camera and Leica LAS EZ software version 2.0.0, Morrisville, NC, USA. A scale bar was added, and fungal dimensions were measured using ImageJ software [57]. The number of replicates is shown in Table 1.

Petri dishes containing *Aspergillus* isolates were further incubated for two more weeks to study the fungal spore dissemination, and glass lids were photographed. *Aspergillus* isolates were also inoculated into two sets of 250 mL Erlenmeyer flasks containing 100 mL of malt extract broth (MEB, pH 5.4) and potato dextrose broth (PDB, pH 5.6) and incubated on a rotary shaker (180 rpm) at 28 °C for seven days in the dark. Culture filtrates were further used for the evaluation of mycotoxins using HPLC. Digital photography for Petri dishes and Erlenmeyer flasks was performed using a Canon EOS 550D digital camera equipped with a Canon 18–55 mm zoom lens. The collected data were recorded in five consecutive trials with three replicates in each at different time points.

### 2.3. Scanning Electron Microscopy (SEM)

Fungal cultures grown on PDA media for 7 days at 28 °C in the dark were used for SEM photography as per [58,59]. A 5 mm square was excised from fungal cultures and immersed into paraformaldehyde (2%) and glutaraldehyde (2.5%) fixative (pH 7.4) at room temperature for two hours for fixation purposes. Samples were then washed thrice, and post-fixed in 1% osmium tetroxide at room temperature for 1 h. Fungal specimens were then washed three times in phosphate buffer (pH 7), vacuum dried, and mounted to the stub. The samples were then coated with gold using a sputter coater. The fungal specimens were examined and photographed using a scanning electron microscope JOEL, JSM-6510LV at 30 kV and a 10 mm working distance at the Electron Microscopy Unit, Mansoura University, Mansoura, Egypt.

### 2.4. Mycotoxin Analysis

For qualitative detection of aflatoxins B and G, *Aspergillus* isolates were grown on coconut meal agar (CMA) pH 7. Coconut extract broth (CEB) was prepared as described by [60] and 2% agar-agar (Oxoid, Basingstoke, Hampshire, England) was added before autoclaving at 121 °C for 20 min. CMA dishes were incubated at 28 °C for seven days in the dark. Two aflatoxigenic *A. flavus* isolates secreting aflatoxin B and G were used as positive controls. The aflatoxin-positive isolates were kindly provided by the Central Lab of Residue Analysis of Pesticides and Heavy Metals in Food (QCAP Lab), Dokki, Giza, 12311, Egypt. The aflatoxigenic capability was visually examined under ultraviolet (UV) light at a wavelength of 366 nm in a dark room using a handheld UV lamp UVGL-55 multiband, San Gabriel, CA, USA.

Quantitative analysis was carried out using HPLC with fluorescent detection (Knauer, Germany). A 100 μL spore suspension of each isolate was inoculated in PDB (pH 7.0) and incubated on a rotary shaker (200 rpm) at 28 °C for five days. Cultures were filtered through cheesecloth, 11 µm pore size Whatman filter paper no. 1, and centrifuged at 7000× *g* for 10 min. Clarified culture filtrates were used to measure the aflatoxins AFB1, AFB2, AFG1, AFG2, and ochratoxin A (OTA). Aflatoxins were separated in an HPLC column (XBridge C18 5 µm 4.6 × 150 mm) with a mobile phase of toluene: ethyl acetate: formic acid: methanol (90:6:2:2, *v*/*v*/*v*) at a flow rate of 1.5 mL min^−1^. Aflatoxin retention times and fluorescence detection excitation and emission wavelengths for AFB1, AFB2, AFG1, AFG2, and OTA are presented in Table 1. Calibration curves for each aflatoxin were determined using a series of standard solutions prepared in methanol.

### 2.5. Sequence Analysis

A consensus sequence of ten representative strains from *A. japonicus, A. niger*, *A. flavus,* and *A. pseudoelegans* was predicted using the ClustalW algorithm in SeaView 5.0.5 software [61]. Sequences of *Aspergillus* isolates used in the present study were included. GenBank IDs for the retracted sequences were previously published by [37]. The consensus sequences were curated, aligned, and edited using BioEdit software [62]. Sequence alignment infers the single nucleotide polymorphisms (SNPs) among *A. japonicus*, *A. niger*, *A. flavus*, and *A. pseudoelegans.*

### 2.6. Biocontrol Assays

The biocontrol potential of *Aspergillus* isolates on the mycelial growth of *S. sclerotiorum* was evaluated in vitro using the dual culture technique on PDA (pH 5.6). Inoculum of each isolate of *Aspergillus* and the target pathogen were placed at equal distances from the Petri dish periphery at opposing positions and incubated at 23 °C as an optimal temperature for the pathogen and the bioagent.

To further assess the biocontrol potential of *Aspergillus* spp. at a wide range of ambient temperatures, freshly propagate mycelial disks of *S. sclerotiorum* were placed in the center of fresh PDA dishes (pH 5.6). Five microliters from 10^6^ cfu/mL^−1^ *Aspergillus* spore suspension was placed beneath the mycelial disks. All treatments were repeated using sclerotia of *S. sclerotiorum*. Both sets of treatments were conducted twice and incubated at 18 °C and 28 °C until *S. sclerotiorum* growth reached the dish edges in the control treatment. Three replicates were used for each treatment. PDA dishes inoculated with mycelium or sclerotia of *S. sclerotiorum* were used for control. Data were photographed to present the nature of coexistence between the tested fungi.

For in vivo biocontrol assays on detached leaves, the inoculum of the fungal pathogen was placed on freshly harvested healthy-looking leaves kept in moisten 15 cm Petri dishes under aseptic conditions. Mycelial disks and sclerotia of fresh *S. sclerotiorum* cultures were used for inoculation purposes. Ten microliters of 1 × 10^6^ cfu/mL^−1^ *Aspergillus* spore suspension was applied beneath the inoculum of the fungal pathogen. Leaves inoculated with *S. sclerotiorum* only were used as a positive control, while *Aspergillus*-inoculated leaves were used as negative controls. Dishes were incubated in a growth chamber at a temperature range of 22–25 °C under a 16/8 h day/night cycle using 60-Watt GE cool white fluorescent bulbs. The mean diameter of the infected zone was measured after one week of incubation, and leaves were photographed.

Two greenhouse experiments were performed at the Faculty of Agriculture, Ash-Sharqiah (GPS coordinates: 30.574947607298547, 31.539601650127825) and Agriculture Research Center, Giza governorates (GPS coordinates: 30.02087346472314, 31.20777383638277) in September 2021 to evaluate the biocontrol potential of *Aspergillus* spp. on white mold disease in a pot experiment. Common bean seeds were dressed with 1 × 10^6^ cfu/mL^−1^ fungal spore suspension at a ratio of 10 µL per seed with the aid of Arabic gum as described in [63]. Seeds were air-dried and stored in a dark dry cabinet for further use. Three seeds were sown in pots filled with peatmoss: vermiculite potting medium pre-infested with 1% of *S. sclerotiorum* inoculum grown on sorghum seeds. Inoculum preparation and soil infestation was carried out as per [37,64]. Untreated seeds were sown in *S. sclerotiorum*-infested soil and virgin soil to serve as a negative control and mock treatment, respectively. An additional control treatment having bean seeds dressed with 50% iprodione (WP) and sown in *S. sclerotiorum*-infested soil was included for positive control comparison. Pots were watered and fertilized when needed. Five pots were used for each treatment.

Data were recorded after two months of seed sowing. Plants were visually inspected for the presence of white mold symptoms, and disease incidence (DI) was calculated as the percentage of symptomatic plants within each treatment. The amount of disease severity within each treatment was assessed according to the following scale: 0 = no diseased stem; 1 ≤ 5%; 3 = 6–15%; 5 = 16–30%; 7 = 31–50%; and 9 ≥ 50% [65]. The disease severity index was calculated using the formula: DS = ∑(nv)/NV × 100, where n is the number of treated plants at the rate v, v is the rate of disease as represented in the disease scale, N is the total number of treated plants in each treatment, and V is the highest disease severity rate in the disease scale. Afterward, biocontrol efficiency was calculated using the formula: Control efficacy = (the mean disease index of the control − the mean disease index of a treatment)/the mean disease index of the control × 100%.

### 2.7. Data Analysis

Data were analyzed in a one-way analysis of variance (ANOVA) test and plotted into histograms using GraphPad Prism version 9.3.1 for Windows, GraphPad Software, La Jolla, CA, USA. Post hoc analysis was conducted using Tukey’s honestly significant difference (HSD) test for pairwise comparison among treatments. Statistical analysis was performed at a significance level where the alpha value = 0.05 and letters on the bar graph indicate significance between different groups.

## 3. Results

### 3.1. Polyphasic Characterization of Aspergillus Species

**Figure 1:**
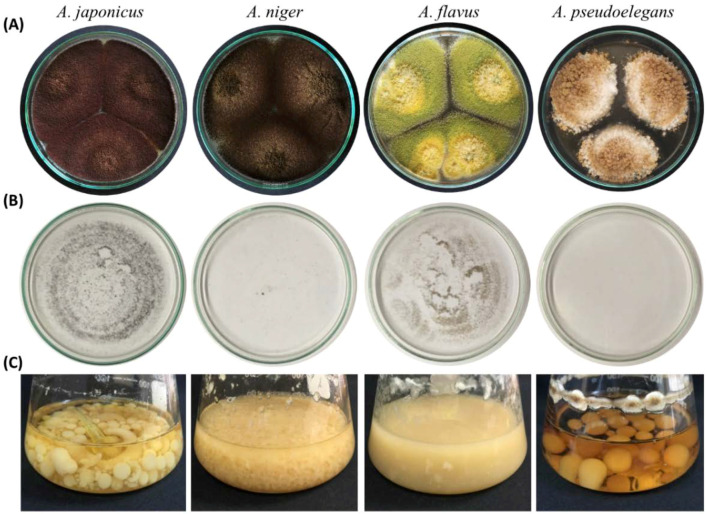
Morphological characterization of *Aspergillus japonicus*, *A. niger*, *A. flavus*, and *A. pseudoelegans*. (**A**) Three-point inoculation for colonies of *Aspergillus* species on MEA after 7 days of incubation at 28 °C. (**B**) Glass lids of dishes containing *Aspergillus* colonies showing spore dust. (**C**) Mold-like growth of *Aspergillus* spp. MEB was incubated on a rotary shaker (200 rpm) at 28 °C for seven days.

**Figure 2:**
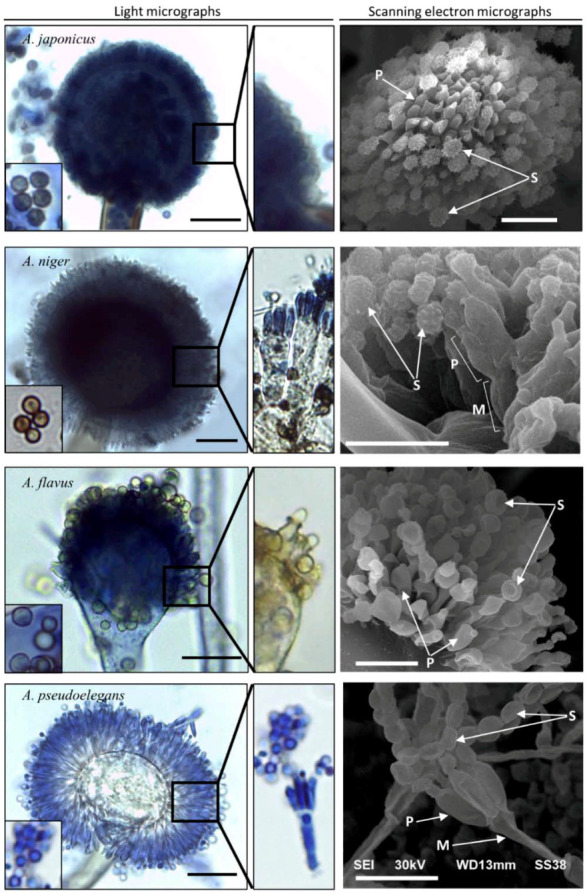
Light and scanning electron microscopic characterization of *Aspergillus* spp. Spores (S) are represented in the small bottom left panel in each photo. Phialides (P) and seriation types are shown in each side panel under higher magnification power. Metulae (M) is indicated in biseriate fungal heads. The scale bar is 10 µm in both light and electron micrographs.

Four morphologically distinct isolates of *Aspergillus* designated *A. japonicus* Eg-F19Asp101 (GenBank ID: MK909934), *A. niger* Eg-F19Asp102 (MK909932), *A. flavus* Eg-F19Asp103 (MK909933), and *A. pseudoelegans* Eg-F19Asp104 (MK909935) were characterized based on their macroscopic and microscopic features on malt extract agar (MEA) medium at 28 °C after 7 days of inoculation. The aflatoxigenic and phytopathogenic capabilities of each isolate were investigated. The collected species showed variability in colony morphology (Figure 1 and Table 2), conidiophore seriation, spore size, phialide length, vesicle shape, and size (Figure 2 and Table 3).

#### 3.1.1. Microscopic Features of *Aspergillus* spp.

**Figure 3:**
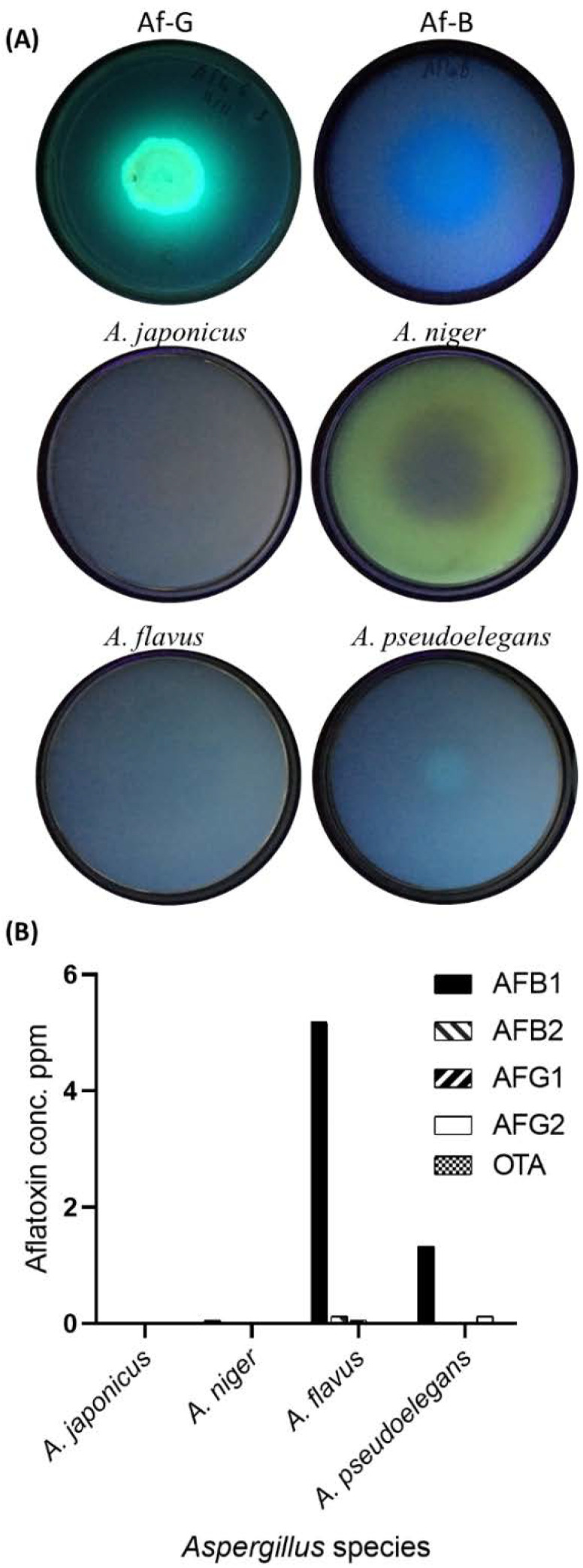
Detection of aflatoxin secretions by *Aspergillus* spp. (**A**) Qualitative detection of aflatoxins in *Aspergillus* cultures grown on coconut meal agar (CMA) medium using UV wavelength of 366 nm. Two green (AflaG) and blue (AflaB) aflatoxigenic *Aspergillus flavus* were used as a positive control. Four species of *Aspergillus* designated *A. japonicus*, *A. niger*, *A. flavus*, and *A. pseudoelegans* were examined. (**B**) Quantitative detection of mycotoxins in *Aspergillus* cultures using HPLC. Aflatoxins AFB1, AFB2, AFG1, AFG2, and OTA were measured in µg/mL (parts per million: ppm).

*A. japonicus* formed coffee brown colonies on MEA with 85 mm diameter after seven days of incubation at 28 °C (Figure 1). The fungus has radiated uniseriate heads with globose to ellipsoidal vesicles (Figure 2). *A. niger* colonies were dark brown, and their diameter reached 90 mm after 7 days (Table 2). *A. niger* heads were biseriate with globose vesicles. The reverse color of both fungi was similar on MEA (Figure S1). However, the bottom side of *A. niger* colonies on potato dextrose agar (PDA) medium was pale yellow circled in a white halo of mycelium. A similar observation was noticed on *A. niger* colonies under UV light (Figure 3). The reverse color was glowing yellow fluorescent light. Aged cultures of both black *Aspergilli* formed black rounded shells surrounding their heads after a prolonged period of incubation (Figure S2). This black rounded structure was frequently observed in *A. japonicus* cultures while it was very rare in *A. niger* cultures. This observation reappeared in five consecutive trials. Mean diameters of vesicles and conidia for each tested *Aspergillus* isolate are represented in Table 3. The variable morphological topology of *Aspergillus* spores, phialides, and heads was clearly illustrated by scanning electron micrographs (Figure 2, right panel). Spores of *A. japonicus* and *A. niger* were spherically shaped with a rough surface covered with short spikes. Spores of *A. niger* were born on a biseriate metulae.

Colonies of *A. flavus* and *A. pseudoelegans* were yellow green and light brown, respectively. *A. flavus* had uniseriate conidiophores, while *A. pseudoelegans* heads were biseriate. The mean diameter of *A. flavus* colonies was 90 mm after 5 days of incubation on MEA, while the diameter of *A. pseudoelegans* colonies was 50 mm after 7 days (Table 3). Conidial heads of *A. flavus* were radiate with sub-spherical to ellipsoidal-shaped vesicles, while *A. pseudoelegans* vesicles were globose. Scanning electron micrographs showed cup-shaped and smooth conidiospores of both *A. flavus* and *A. pseudoelegans* born on uniseriate and biseriate phialides, respectively. Macro and micromorphological characters of the examined isolates confirmed their identity and emphasized their variability [52,66,67]. *Aspergillus* spp. exhibited variable growth rates and temperature preferences.

Noticeably, the uniseriate fungal isolates *A. japonicus* and *A. flavus* discharged a dense cloud of spores on the glass lid of Petri dishes while the biseriate isolates, *A. niger* and *A. pseudoelegans,* did not (Figure 1B). Sporulation of all *Aspergillus* isolates was absent at 18 °C under constant dark conditions (Table 2 and Figure S3). No sclerotia, cleistothecia, fungal exudates, or soluble pigments were visually detected on MEA, PDA cultures, or malt extract broth (MEB) of the tested *Aspergillus* spp. (Figure 1 and Figure S1), except that the reverse color of *A. japonicus* had a yellow hue. In addition, potato dextrose broth (PDB) culture inoculated with *A. pseudoelegans* was orange to light brown at seven days post-incubation (dpi) on a rotary shaker at 180 rpm (Figure S1C). The growth pattern of *Aspergillus* spp. was clearly variable on MEB only in terms of colonies’ shape and size (Figure 1C).

#### 3.1.2. Mycotoxin Secretions by *Aspergillus* spp.

Mycotoxin secretion by *Aspergillus* spp. was evaluated qualitatively under ultraviolet (UV) light and quantitively by HPLC. Two aflatoxigenic species of *A. flavus* were used as a control for the qualitative detection of aflatoxin B and G, respectively. No blue or green fluorescent was detected on *Aspergillus* cultures grown on coconut meal agar (CMA) (Figure 3). Likewise, no aflatoxin or ochratoxin A production was detected by HPLC for the examined isolates except for *A. flavus*, which produced 5 ppm of aflatoxin B1, and *A. pseudoelegans* that produced 1 ppm of the same toxin (Figure 3B, Figures S4 and S5). Plant pathogenic capability of the examined *Aspergillus* spp. was also examined on detached leaves of common bean, soybean, cowpea, and tobacco. *Aspergillus* spp. were unable to induce any visual symptoms on plant leaves after two weeks of incubation under humid conditions. Those observations infer that examined *Aspergillus* species were not plant pathogenic but two of which were aflatoxigenic.

#### 3.1.3. Genetic Variations within *β-tubulin* of *Aspergillus* spp.

Multiple sequence alignment (MSA) of partial *β-tubulin* sequences from *Aspergillus* spp. under this study was carried out to study the variations among the tested isolates. Multiple nucleotide insertion, deletion, and substitution were detected throughout the alignment (Figure S6). However, no single pattern discriminating the four species was observed. Several isolate-specific single nucleotide polymorphisms (SNPs) were detected throughout the sequences such as A24, A57, G58, T60, G61, C62, T64, C65, G71, C74, G75, G107, G133, A134, C137, T145, T154, G258, C276, A357, and A364 in the consensus sequence of *A. flavus*. Similarly, common SNPs discriminating the two black *Aspergilli* isolates were detected at positions C55, G81, C83, A155, G156, C157, C161, and C516. The majority of variations detected in the alignment were lying at the 5′-proximal third of the consensus sequences.

### 3.2. Aspergillus Deteriorates the Mycelium of S. sclerotiorum and Colonizes Its Sclerotia

**Figure 4:**
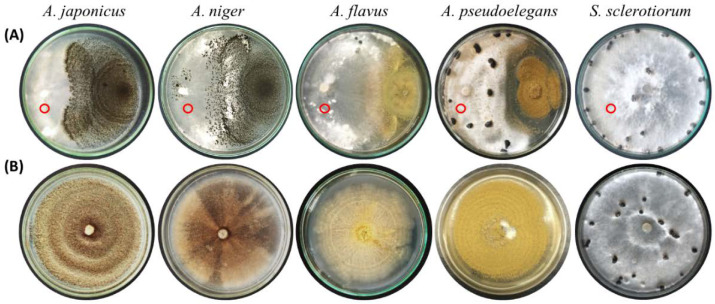
Antagonistic interaction between *Aspergillus* spp. and *S. sclerotiorum* in vitro. Inoculum of four *Aspergillus* spp. and *S. sclerotiorum* placed on PDA medium at opposing positions of Petri dish periphery and incubated at 28 °C for one week (**A**). Dishes solely inoculated with *S. sclerotiorum* represent the control treatment. Mycelial disks of the pathogen’s colony, indicated by red circles, were transferred to new PDA dishes and incubated at 28 °C for one week (**B**).

The ability of *Aspergillus* spp. to parasitize sclerotia of *S. sclerotiorum* and prevent its germination was previously confirmed. The inhibitory effect of *Aspergillus* spp. on *Sclerotinia’s* mycelial growth was examined using dual culture technique. At seven days of inoculation, 5 mm mycelial disks of *Aspergillus*-infested *S. sclerotiorum* colonies were transferred to fresh PDA dishes (pH 5.6) and incubated for one week. Mycelium of *S. sclerotiorum* was unable to grow as *Aspergillus* spp. purely dominated the entire culture (Figure 4). No pigments were observed in examined dishes except for *A. flavus*, which turned the reverse color to orange-brown and decayed the existing mycelium of *S. sclerotiorum*. To visualize the interaction between *Aspergillus* and *Sclerotinia*’s mycelia in dual culture, we examined the intermingled region of mycelia microscopically using the slide culture technique. No signs of physical interaction or contact were observed between the two mycelia as they grew at separate levels of the substrate. These results inferred the competition potential of *Aspergillus* spp. in dual cultures against *S. sclerotiorum*.

### 3.3. Aspergillus spp. Retain Their Biocontrol Potential at a Wide Range of Temperature

**Figure 5:**
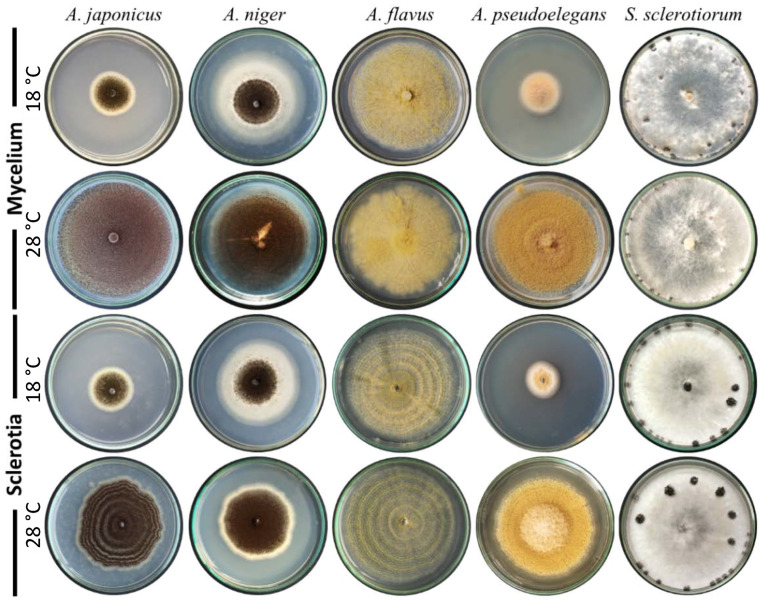
Biocontrol potential of *Aspergillus* spp. against mycelium and sclerotia of *S. sclerotiorum* at a wide range of ambient temperatures. Spore suspension of *Aspergillus* spp. was added to the culturing media beneath the mycelium disks or sclerotia of *S. sclerotiorum*, and incubated at 18 °C or 28 °C.

The role of temperature on successful interaction between *Aspergillus* spp. and *S. sclerotiorum* was studied in vitro. Both fungi were co-inoculated in the center of fresh PDA dishes and incubated at 18 and 28 °C as favored by the pathogen and the potential bioagent, respectively. Growth and reproduction of *S. sclerotiorum* were observed only in the control treatments where no *Aspergillus* spp. were inoculated (Figure 5). Growth and sporulation patterns of *A. japonicus*, *A. niger,* and *A. pseudoelegans* were slightly altered under low temperatures. However, *Aspergillus* spp. were able to dominate and cover the entire substrate of all other treatments under the examined temperatures and significantly suppress *Sclerotinia’s* growth.

### 3.4. Aspergillus Suppresses White Mold Disease on Legumes

**Figure 6:**
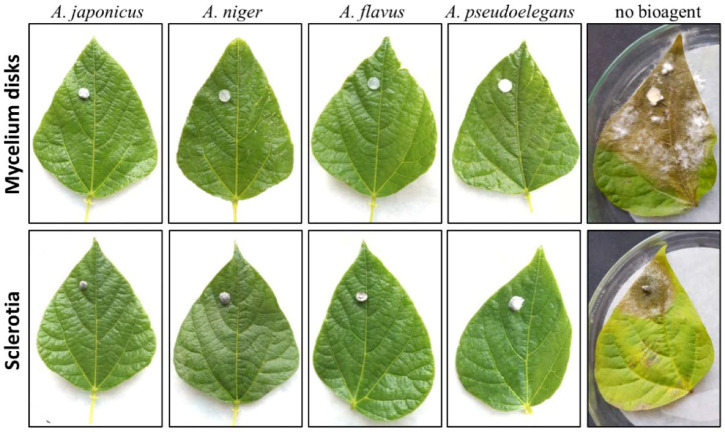
Biocontrol potential of *Aspergillus* spp. against white mold disease on detached common bean leaves. Detached leaves from healthy common bean plants inoculated with freshly propagated *S. sclerotiorum* inoculum. Two types of inoculums were used: mycelial disks and surface sterilized sclerotia. Inoculum of each *Aspergillus* spp. was introduced beneath the inoculation point. Treatments were incubated in a growth chamber at a temperature range of 22–25 °C under a 16/8 h day/night cycle using 60-Watt GE cool white fluorescent bulbs. Inoculated leaves were photographed after 7 days of inoculation. Representative pictures of one replicate; other two replicates showed the same phenotype.

The ability of *Aspergillus* spp. to suppress white rot disease in vivo was further assessed on detached leaves of common bean, soybean, and cowpea. The leaves were separately inoculated with mycelial disks and sclerotia of *S. sclerotiorum*. Typical white mold symptoms were developed on leaves inoculated with *S. sclerotiorum* alone. Water-soaked lesions started to develop around the inoculation point, then the symptoms rapidly progressed and turned brown as they expand. Infected tissue was covered with a white fluffy mycelium when relative humidity was high. Fungal mycelium turned into black sclerotia after four to five days of symptoms development. No signs of infection were observed on detached leaves co-inoculated with *S. sclerotiorum* and *Aspergillus* spp. (Figure 6 and Figure S7, Table 4).

**Figure 7:**
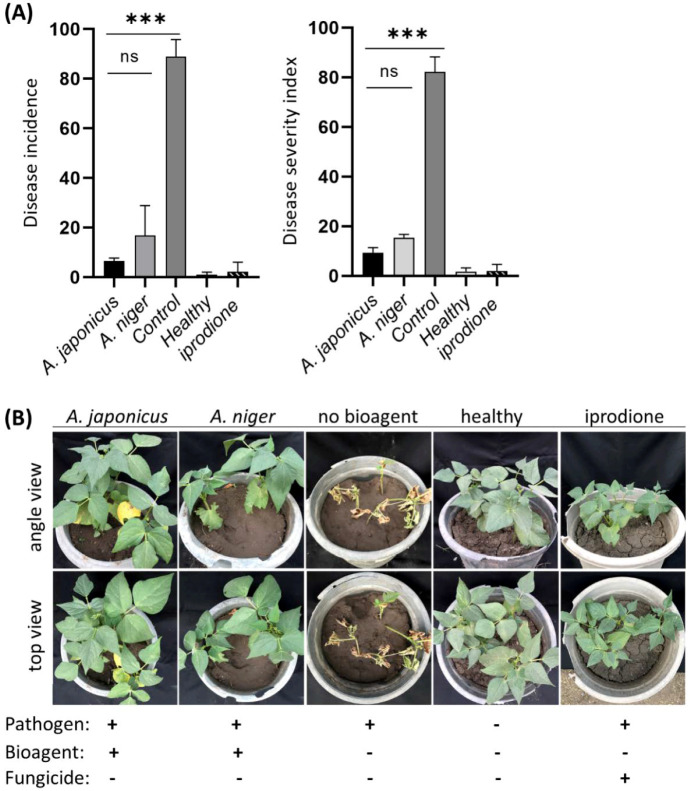
Biocontrol potential of *Aspergillus* spp. on white mold disease under greenhouse conditions. Bean seeds dressed with *A. japonicus* and *A. niger* spores, sown in *S. sclerotiorum*-infested soil were scouted for visual symptoms after one month of sowing. (**A**) Disease incidence (DI) and disease severity index (DS) percentages of white mold disease on *Aspergillus*- or iprodione-treated and untreated beans. Results were statistically analyzed using one-way ANOVA followed by Tukey’s post hoc HSD test. Values are the mean of five independent replicates and vertical bars are the standard deviations. Asterisks (***) denote significance between treatments and untreated control at *p* = 0.05, while (ns) refers to the lack of significance among treatments. (**B**) Symptomatic beans suffering white mold disease versus healthy and bioagent-treated bean plants. Representative pictures of one replicate; other two replicates showed the same phenotype.

The biocontrol potential of *Aspergillus* spp. was further evaluated on common bean plants in a pot experiment under greenhouse conditions. Plants treated with *A. japonicus* and *A. niger* exhibited significantly lower disease incidence and disease severity percentages than the negative control treatment (Figure 7A). Plants in the control treatment were able to survive infested soil and develop normal seedlings. However, seedling growth was arrested, and the canopy was smaller than uninoculated plants. Necrotic lesions were eventually developed on the stems of the negative control plants and caused a gradual death. Similarly, *A. japonicus* and *A. niger*-treated seeds developed a significantly lower disease severity index compared to the negative control. *A. japonicus* gave higher protection and less disease incidence than *A. niger*, but the gap was nonsignificant. The amount of disease protection provided by *A. japonicus* and *A. niger* was slightly lower than the chemical fungicide iprodione. The mass of plant canopy/plant vigor in bioagent-treated seeds was bigger than in healthy and fungicide-treated seeds, based on the number of leaves and leaf area, while the number of branches was similar (Figure 7B, tabulated data not shown).

## 4. Discussion

White mold disease causes substantial losses to legume crops worldwide [1,2,3,4]. As a necrotrophic pathogen, *S. sclerotiorum* secretes an arsenal of pathogenicity factors to prepare plant tissue for successful invasion [4]. After invading host tissues, the pathogen generates solid black bodies of uneven shapes called sclerotia for overwintering [3,4,68]. This overwintering stage is known for its ability to survive harsh environmental conditions and remain dormant and viable in soil and plant debris for years. Sclerotia can also survive strong acids, high alkaline solutions, flood, drought, and high soil temperature, as reviewed in [37]. Those facts have rendered several management strategies ineffective against such pathogens. Therefore, integrated management using multiple techniques including biological control offers a better solution to mitigate the disease intensity. Biocontrol seems to be a useful strategy against the resting phase of *S. sclerotiorum*. Several biocontrol agents deteriorate sclerotia and thus minimize their potential in soil, which in turn decreases disease intensity in fields [16,17,69,70].

Biological control has been used for the management of white mold disease in several crops [18,19,20,21,22]. *A. terreus* and *A. aculeatus* effectively reduced *Sclerotinia* white rot disease [31,32]. In this research, we characterized the biocontrol potential of distantly related *Aspergillus* spp. against white mold disease to examine whether this phenomenon is conserved among members of the genus *Aspergillus*. Beforehand, four morphologically distinct species namely *A. japonicus*, *A. niger*, *A. flavus,* and *A. violaceofuscus* were characterized based on their morphological, microscopic, mycotoxigenic, genetic, and pathological characters. The morphological and physiological attributes of the selected candidates were parallel to their reference strains [66,67,71,72,73]. Although being opportunistic plant pathogens for onion and some grain crops, the tested *A. niger* and *A. flavus* isolates did not cause any signs of disease infection on soybeans and common beans on the leaf assay.

Microscopic observation of *Aspergillus* cultures revealed the biseriate nature of *A. niger* and *A. pesudoelegans*, which is in accordance with [55,73] who illustrated the features of *Aspergillus* members in sections *Nigri* and *Circumdati*. Similarly, the observed uniseriate nature of *A. japonicus* and *A. flavus* was previously described by [73] who found that the uniseriate species belong to the *A. aculeatus* clade, which includes *A. japonicus*. As per [54], *A. flavus* and *A. subflavus* have uniseriate conidiophores. The correlation between conidial discharge and seriation type was clearly observed repeatedly in uniseriate members, which were capable of releasing and accumulating more conidiospores on the glass lid of the Petri dishes. This correlation emphasizes the role of seriation type in conidiospores dispersal for examined isolates of *Aspergillus* spp. To our knowledge, the roles and reasons behind variable seriation types were not previously inferred.

Notably, the isolates of the *Nigri* section formed black round shells around some of its conidial heads after four weeks of incubation at 28 °C on PDA, apparently for long-term protection. Those conidia-filled vesicles are precursors for sclerotia, with sclerotia being an essential prerequisite for sexual state development [74,75]. Sclerotial formation at the apex of conidiophore heads was previously reported by [75]. The presence of sclerotia in *Aspergillus* cultures was previously demonstrated for the homothallic species *A. japonicus* and the heterothallic species *A. niger* [75,76,77].

Each of *A. japonicus*, *A. niger,* and *A. pseudoelegans* had radial furrows on MEA but not PDA. This feature was previously reported for members of sections *Nigri* and *Circumdati* [55,67]. The reverse color of *A. japonicus* was pale yellow on PDA. This observation was also reported for several members of section *Nigri* by [66]. The liquid PDB cultures of *A. pseudoelegans* become orange to light brown in color, which could be attributed to its ability to produce neoaspergillic acids similar to other members of the same section [55]. Aspergillic acid is yellow colored [78], which could be the cause of color change in the growing broth media. No soluble pigments were observed in examined dishes of *Aspergillus* spp. grown on solid media except for *A. flavus*, which turned the reverse color to orange-brown and decayed the existing mycelium of *S. sclerotiorum*. This observation was not detected in pure liquid or agar cultures of *A. flavus*, which indicates that the detected secretion was inducible upon biotic stress/competition. kojic acid and aspergillic acids are among the major secretions of *A. flavus* in growing culture media [79,80]. Both acids are known to possess antifungal and antibacterial activity, which may be responsible for mycelial decay [78,81,82].

The growth rate and sporulation of tested *Aspergillus* spp. were dramatically affected by darkness, particularly under low ambient temperature. Sporulation of biseriate fungal species, *A. niger,* and *A. pseudoelegans*, was more sensitive to light conditions than uniseriate ones. Dark conditions completely halted the sporulation ability of biseriate fungi and delayed it in uniseriate ones. The effect of light on sporulation of *Aspergillus* spp. was previously demonstrated [83,84,85,86,87,88]. The fungal scaffold protein VeA plays an integral role in signal transduction of environmental cues including light. The VeA protein integrates blue and red-light signals and responds by regulating the asexual and sexual reproductive cycle. VeA accumulates in the nucleus and represses asexual development under dark conditions [89]. Recently, Sun et al. [88] discovered the light-activated transcription factor, *RlmA*, in *A. niger*, which is required for the synthesis of melanin, chitin, and exopolysaccharides. Light signaling promotes film production in *A. niger*, while an absence of light or defective light-activated factors yields abnormal colonies bearing less number of conidiophores [88]. The concentric circle growth of *Aspergillus* colonies in Figure 5 and Figure 6 was another phenotypic response to dark/light cycles during incubation.

The biocontrol potential of *Aspergillus* spp. was confirmed through in vitro and in vivo studies. The fungal growth of *Aspergillus* spp. did not show any signs of competition or antibiosis. However, *Aspergillus* spp. growth was able to expand through the opponent’s fungal colony and concomitantly grow asymmetrically. Presence of *Aspergillus* spp. growth within the pathogen’s colonies significantly suppresses its asexual reproduction and vegetative regeneration. We previously showed that disturbance of ambient oxalic acid concentration suppresses sclerotial formation [37]. In addition, *Aspergillus* secretions are suppressive to certain fungal plant pathogens including *S. sclerotiorum* [90,91,92]. Metabolites from *Aspergillus capensis* culture filtrate namely methyl dichloroasterrate, penicillither, and rosellichalasin showed antifungal activity against *S. sclerotiorum,* in vitro [93]. Interestingly, *Aspergillus* spp. were able to maintain their biocontrol potential against *S. sclerotiorum,* in vitro, even at unfavorable temperatures, which makes them a good choice for being sustainable bioagents.

*Aspergillus* spp. also prevented white mold disease onset on soybean and common bean detached leaves inoculated with *S. sclerotiorum* mycelium or sclerotia, which confirms the biocontrol potential of *Aspergillus* spp. In the greenhouse experiment, *A. flavus* and *A. pseudoelegans* were avoided because of their mycotoxigenic secretions detected by HPLC. The ability of *A. japonicus* and *A. niger* to suppress white mold disease on common beans grown in *S. sclerotiorum*-infested potting media was comparable to the commercially available synthetic fungicide iprodione WP (50%) Bayer, Germany. Moreover, plant vigor and canopy density were much bigger in *Aspergillus*-treated plants, which suggests the supportive roles of both black *Aspergilli* as endophytes in legume plants. The endophytic potential of *A. japonicus* and *A. niger* was previously proven [90,91,92,94,95,96]. The pronounced ability of black *Aspergilli* in producing a high amount of oxalic acid and enhancing plant vigor has sparked the idea to investigate their endophytic growth in different legumes, which is currently ongoing in our lab.

## 5. Conclusions

In conclusion, *Aspergillus* species have beneficial mycoparasitic capability against white mold disease induced by *S. sclerotiorum*. The examined species of *Aspergillus* were morphologically, microscopically, genetically, and physiologically distinct, but they all share the ability to suppress white mold disease. In essence, *Aspergillus* spp. inhibited *Sclerotinia’s* germination and deteriorated its mycelium in vitro. *Aspergillus* spp. were also protective for common bean and soybean detached leaves inoculated with *S. sclerotiorum*. The non-mycotoxigenic species, *A. japonicus* and *A. niger*, were able to reduce the severity and incidence of white mold disease in the greenhouse. Some characteristic features of *Aspergillus* spp. were also revealed in this study, such as the head shells that distinguish black *Aspergilli*. The role of seriation type in conidiospore discharge was also suggested. It was noticed that uniseriate *Aspergilli* discharge more spores than biseriates. Sporulation of *Aspergillus* spp. is light dependent, but to different levels. The absence of light completely abolishes sporulation of the biseriates *A. niger* and *A. pseudoelegans*, but delays sporulation of the uniseriates *A. japonicus* and *A. flavus*.

## Figures and Tables

**Table 1:** Parameters of HPLC analysis of aflatoxins AFB1, AFB2, AFG1, AFG2, and ochratoxin A. Mycotoxin retention times at 1.5 mL/min. flow rate were calculate in minutes. The excitation and emission wavelength are presented in nanometers.

HPLC Parameters	AFB1	AFB2	AFG1	AFG2	OTA
Retention time (min)	5.1–7.1	12.9–13.2	5.2–5.5	8.7–9	6.7
Excitation Wavelength	365	365	365	365	335
Emission Wavelength	450	450	450	450	465

**Table 2:** Macroscopic characters of *Aspergillus* isolates. Colony color was described for individuals grown on malt extract agar (MEA) medium after 7 days post inoculation (dpi). All other presented features were monitored for isolates grown on potato dextrose agar (PDA).

	Colony Color	Colony Diameter at 28 °C/7 dpi	Sporulation at 18 °C/Dark	Seriation Type	Spore Liberation	Sclerotia on PDA
*A. japonicus*	Coffee brown	90 mm	Normal	Uniseriate	Dense	Absent
*A. niger*	Dark brown	90 mm	Absent	Biseriate	Light	Absent
*A. flavus*	Yellow green	90 mm at 5 dpi	Normal	Uniseriate	Dense	Absent
*A. pseudoelegans*	Light brown	50 mm	Absent	Biseriate	Light	Absent

**Table 3:** Microscopic dimensions of *Aspergillus* isolates. *Aspergillus* heads, vesicles, spores, conidiophores, and phialides measured in micrometers (µm). Photos were taken by a Leica DM500 compound microscope and processed using ImageJ software. Scanning electron micrographs were also analyzed to reassure the microscopic dimensions. Number of examined replicates is shown in the bottom row of the table.

	Head Diameter (µm)	Vesicle Diameter (µm)	Spore Diameter (µm)	Conidiophore Width (µm)	Phialide Length (µm)	Spore Surface
*A. japonicus*	33.9	25.2	2.11	7.2	4.23	Rough
*A. niger*	38.8	20.6	1.606	6.7	4.5	Rough
*A. flavus*	25.8	18.1	2.64	4.8	3.9	Smooth
*A. pseudoelegans*	40.2	17.4	1.343	5.2	5	Smooth
No. of replicates	50	50	100	50	50	100

**Table 4:** Biocontrol efficiency of *A. japonicus* and *A. niger* on white mold disease under greenhouse conditions. Statistical analysis is denoted in Figure 7. Disease incidence (DI) and disease severity (DS) were recorded at two months post seed sowing.

	DI	DS Index	Biocontrol Efficiency
*A. japonicus*	6.5	9.3	88.5
*A. niger*	16.8	15.3	81.4
Control	88.9	82.1	N/A
Healthy	1.0	1.7	N/A

## Data Availability

The data collected and analyzed throughout the present research are available upon request. Fungal sequences were deposited in NCBI GenBank and the accession numbers were given in the text. Supplementary Materials are available on journal website.

## Supplementary Materials

The following supporting information can be downloaded at: https://www.mdpi.com/article/10.3390/jof8060626/s1, Figure S1. Morphological characterization of *Aspergillus japonicus*, *A. niger*, *A. flavus*, and *A. pseudoelegans*. (A and B) the reverse color of *Aspergillus* species on MEA and PDA after 7 days of incubation at 28 °C. (C) Mold-like growth of *Aspergillus* spp. PDB was incubated on a rotary shaker (200 rpm) at 28 °C for seven days. Figure S2. Head shell of black *Aspergilli*. Old cultures of *Aspergillus* spp. of *Nigri* section. *Aspergillus* heads are covered in black rounded hard shells that break open after tapping the glass cover with a glass rod. Scale bar = 100 µm. Figure S3. Mycelial growth of *Aspergillus japonicus, A. niger*, *A. flavus,* and *A. pseudoelegans* at low ambient temperature under dark conditions. (A) The upper color of *Aspergillus* colonies grown at 18 °C. (B) the reverse color of *Aspergillus* colonies. Figure S4. Chromatograms of aflatoxins. A typical chromatogram of the standard solution for aflatoxins is shown (E). Peaks representing aflatoxins AFB1, AFB2, AFG1, and AFG2 are shown. HPLC analysis of organic acids in the culture filtrate of four *Aspergillus* isolates (A, B, C, and D) at 7 days of incubation on PDB (pH 7.0). The corresponding known and unknown peaks detected in the culture medium are indicated on each chromatogram. Figure S5. Chromatograms of ochratoxin A. A typical chromatogram of the standard solution for ochratoxin A is shown (D). Figure S6. Multiple sequence alignment of consensus *Aspergillus* spp. sequences. Alignment was performed using ClustalW algorithm. Sequence alignment infers the single nucleotide polymorphisms (SNPs) among *A. japonicus*, *A. niger*, *A. flavus*, and *A. pseudoelegans.* Figure S7. Biocontrol potential of *Aspergillus* spp. against white mold disease on detached soybean leaves. Detached leaves from healthy soybean plants inoculated with freshly propagated *S. sclerotiorum* inoculum. Two types of inoculums were used: mycelial disks and surface sterilized sclerotia. Inoculum of each *Aspergillus* spp. was introduced beneath the inoculation point. Treatments were incubated in a growth chamber at a temperature range of 22–25 °C under a 16/8 h day/night cycle using 60-Watt GE cool white fluorescent bulbs. Inoculated leaves were photographed after 7 days of inoculation.
