# Supplementary figures and images for: Polyphasic Characterization of Four Aspergillus Species as Potential Biocontrol Agents for White Mold Disease of Bean
Source: J Fungi (Basel). 2022 Jun 12;8(6):626. doi: 10.3390/jof8060626 (PMC9224856; doi:10.3390/jof8060626)

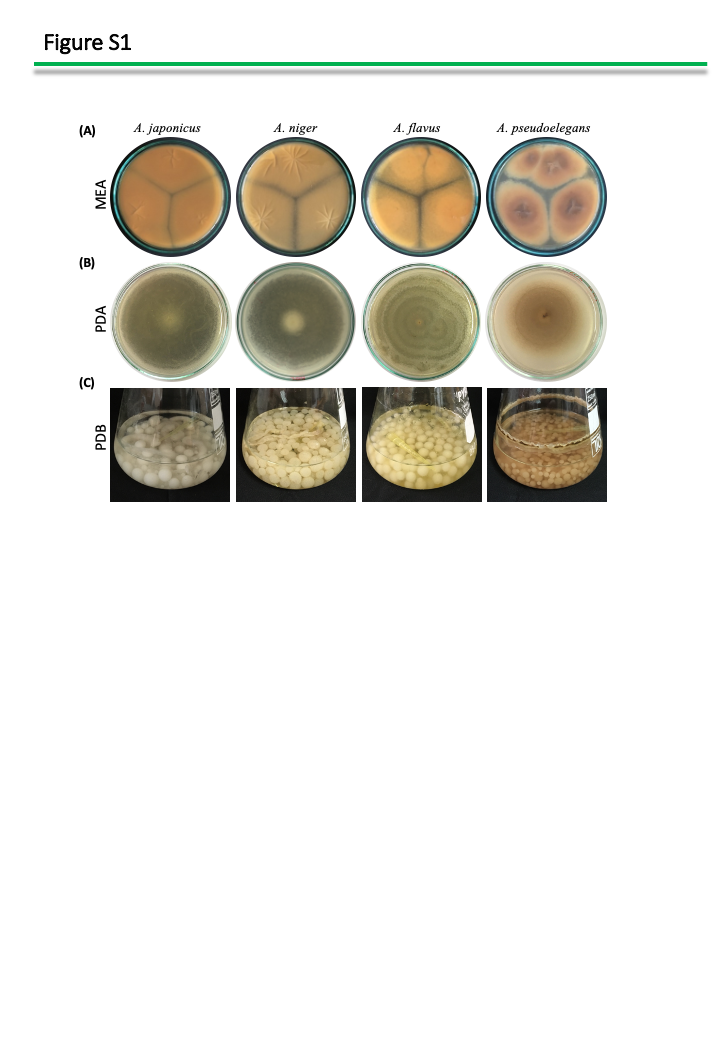

Supplement: Supplementary file 1 [file jof-08-00626-s001.zip › Figure S1.tiff]

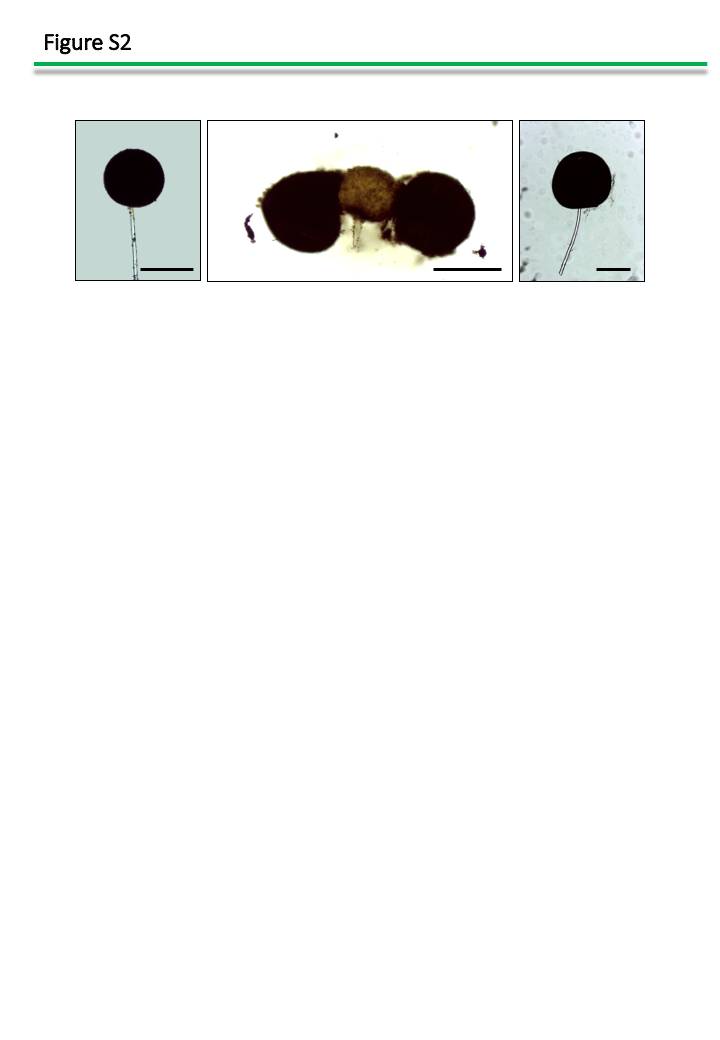

Supplement: Supplementary file 1 [file jof-08-00626-s001.zip › Figure S2.tiff]

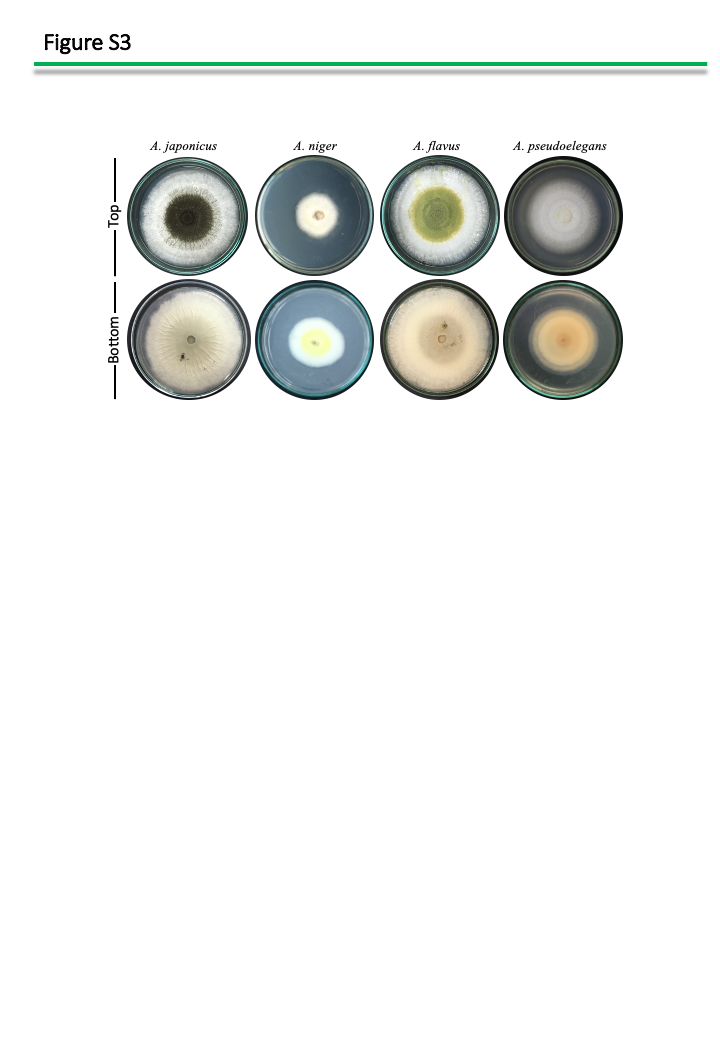

Supplement: Supplementary file 1 [file jof-08-00626-s001.zip › Figure S3.tiff]

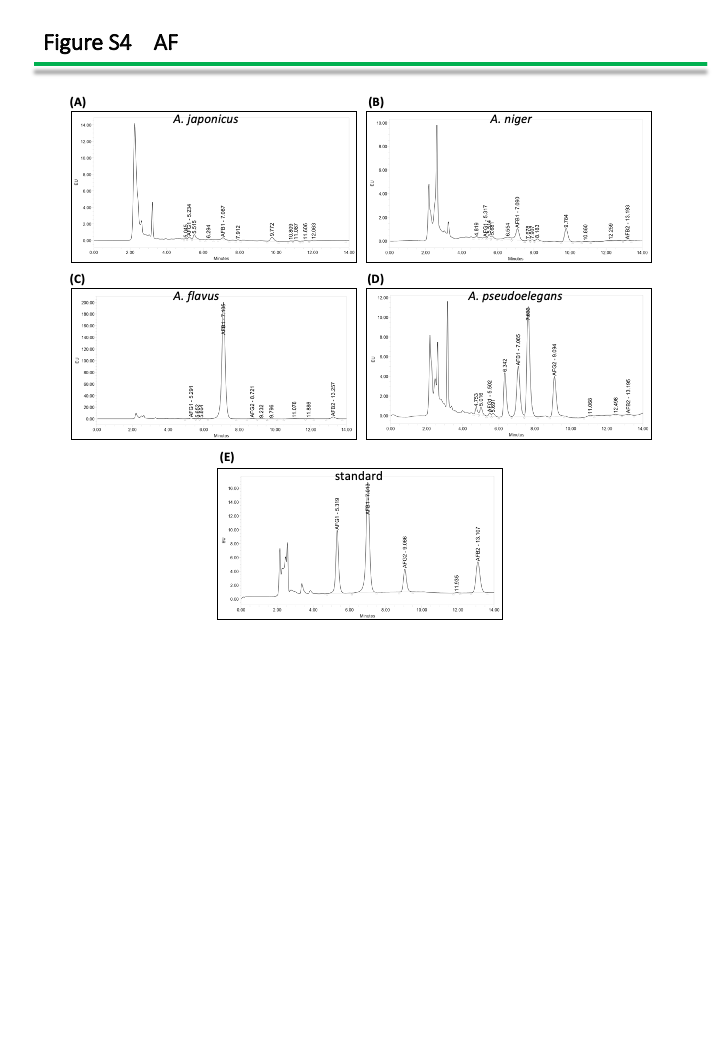

Supplement: Supplementary file 1 [file jof-08-00626-s001.zip › Figure S4.tiff]

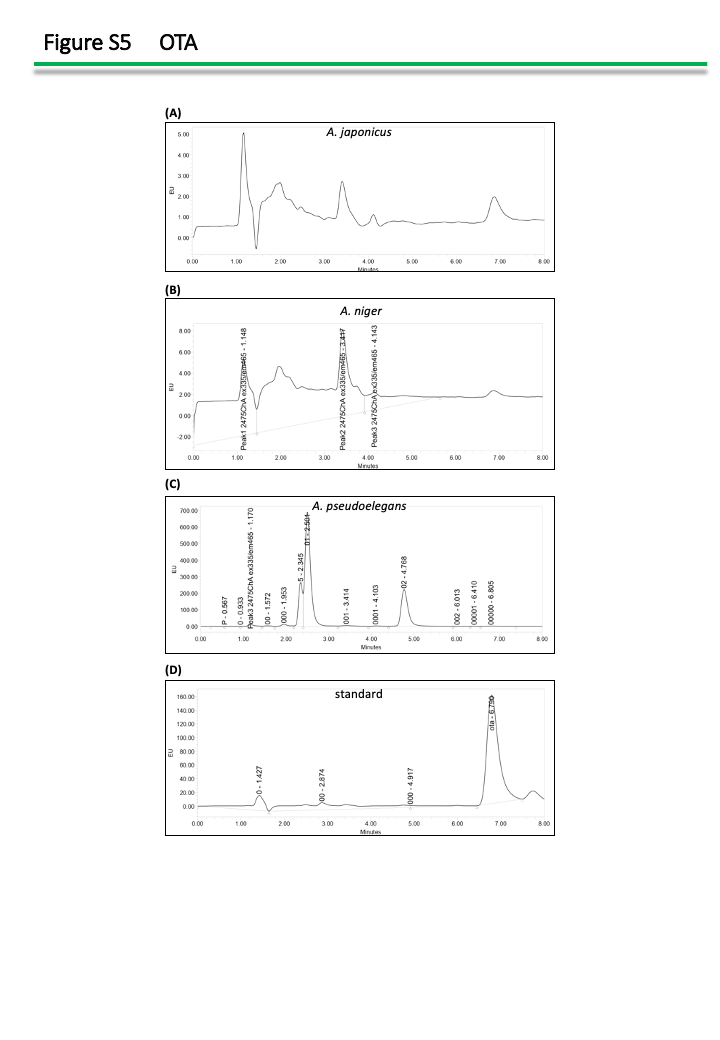

Supplement: Supplementary file 1 [file jof-08-00626-s001.zip › Figure S5.tiff]

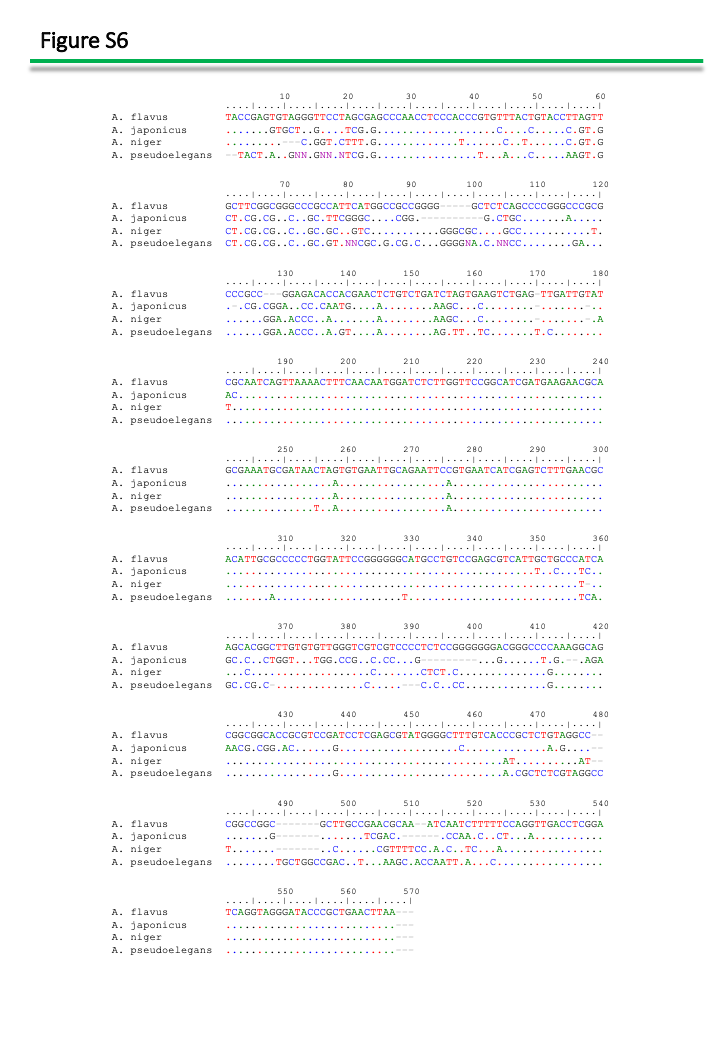

Supplement: Supplementary file 1 [file jof-08-00626-s001.zip › Figure S6.tiff]

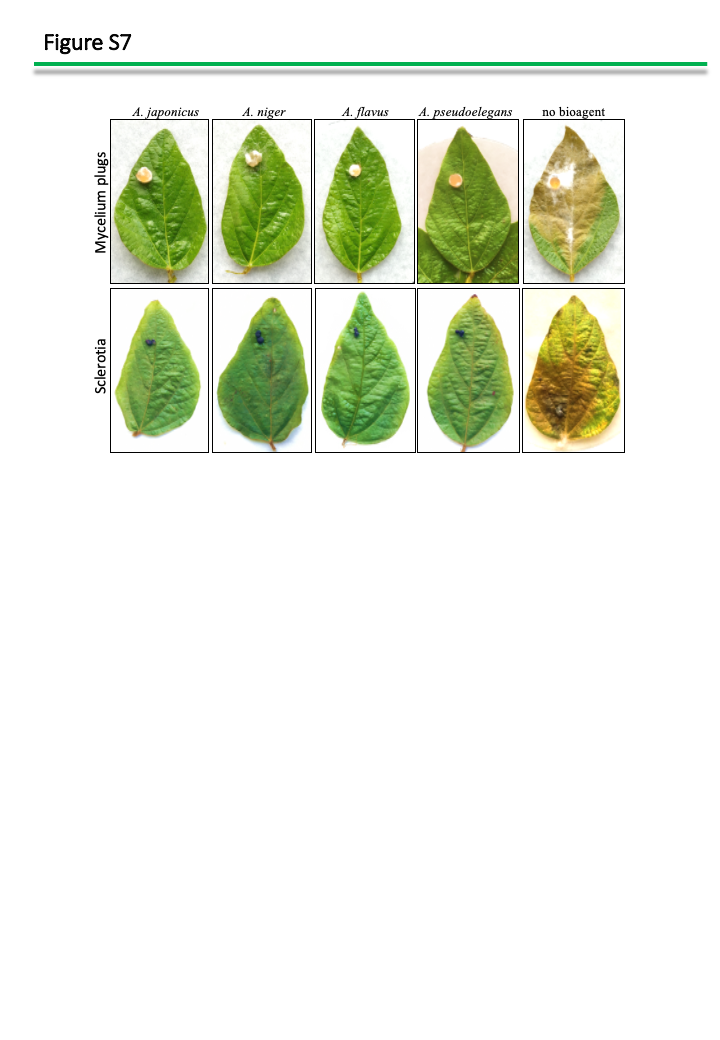

Supplement: Supplementary file 1 [file jof-08-00626-s001.zip › Figure S7.tiff]
